# Embedding of taggants in the vat photo-polymerisation additive manufacturing process for anti-counterfeiting measures

**DOI:** 10.1007/s40964-026-01766-w

**Published:** 2026-05-22

**Authors:** Bochuan Liu, Paula F. Wilson, Mark A. Williams, Gregory J. Gibbons

**Affiliations:** Warwick Manufacturing Group (WMG), University of Warwick, Coventry, UK

**Keywords:** Additive manufacturing, Vat photo-polymerisation, Anti-counterfeiting, Taggant, X-ray computed tomography, Double-lock hash

## Abstract

The rapid expansion of the additive manufacturing (AM) industry has attracted significant global attention. However, the inherent nature of AM, specifically the direct translation of CAD data into physical components, makes the supply chain highly vulnerable to fraudulent and counterfeit activities. As scanning and reverse-engineering technologies advance, existing authentication methods have become increasingly susceptible to replication, and consequently, unreliable. This article investigates a novel authentication method for the vat photo-polymerisation process (VPP) by introducing a “double-lock” system. This system embeds both a physical hash (randomly distributed taggants acting as physically unclonable functions) and a digital hash (derived from X-ray computed tomography scans) into each component. Several digital hashing methods are proposed, including a combination of taggant spatial coordinates, colour codes, and quadrat counts, creating a signature that demonstrates substantial spatial complexity and resistance to physical replication. The chi-square (χ^2^) value of the distribution can also be utilised as a supplemental digital hash. The study indicates that the embedded physical taggants had no discernible effect on the thermal properties of the parts, and exerted minimal impact on mechanical strength. Specifically, reductions in ultimate tensile strength (UTS) at room temperature were limited to 3.1 ± 0.8 %, 3.9 ± 1.1 %, and 1.0 ± 1.0 % for layer thicknesses of 100, 50, and 25 μm, respectively. While the UTS for all samples decreased with increasing temperature, the presence of taggants did not affect the UTS beyond the standard margin of error across most testing conditions. The exceptions were samples tested at 23 °C and 180 °C, which exhibited UTS variations of 4.0 ± 1.3 % and 17.4 ± 3.8 %, respectively, between samples with and without taggants. Overall, the double-lock system developed in this study maintains a high level of security with minimal sacrifice to the components’ thermal and mechanical properties.

## Introduction

Additive manufacturing (AM), also known as 3D printing, has gained significant interest from both industry and academia [1]. Since the 1980s, a broad spectrum of AM technologies has been developed [2]. Among these, vat photo-polymerisation (VPP) has become one of the most prominent techniques, attracting attention across a diverse range of fields, including medicine and dentistry, biomedical engineering, metamaterials, injection moulding, jewellery, and consumer products [3–6]. The fundamental principle of VPP is the layer-by-layer curing of a photosensitive liquid resin to fabricate a solid component [7].

The rapid adoption of AM technology, however, has introduced an undesirable side effect: counterfeit parts have become significantly easier to produce, provided malicious actors possess the necessary computer-aided design (CAD) models and 3D printers [8]. The proliferation of low-cost, easily accessible 3D printers has drastically lowered the barrier to entry for intellectual property (IP) theft and product counterfeiting [9]. Unlike traditional manufacturing, where physical tooling or equipment need to be stolen or reverse-engineered, an AM counterfeiter only needs to intercept a digital file. Cloud-based AM workflows are inherently vulnerable to cybersecurity threats, allowing attackers to steal, copy, or subtly tamper with design files before they even reach the printer [10]. Furthermore, the widespread availability of high-resolution 3D scanners allows counterfeiters to digitise legitimately acquired parts, generating highly accurate models. These can then be used to produce physical clones that are visually identical to the original, often bearing the same surface-level serial numbers [11]. In more sophisticated scenarios, attackers can reconstruct proprietary designs simply by observing a 3D printer in operation. Optical side-channel attacks utilise video analysis of a printhead’s frame-by-frame movements to reverse-engineer exact coordinates and toolpaths, effectively stealing IP without ever infiltrating the host computer [12]. These vulnerabilities within the AM supply chain can result in the premature failure of substandard counterfeit components, leading to severe reputational, brand, and financial damage for the original manufacturer [13].

Consequently, extensive research has focused on developing methods to embed various security features directly into AM components. Over the years, the literature has highlighted a diverse array of strategies, including nanoscale fluorescence printing for 3D labels [14], embedded material-responsive fingerprints [15], wireless and chipless RFID solutions [16–18], and advanced optical and photonic encoding techniques [19–23]. The integration of smart materials, such as luminescent perovskites, quantum dots, and shape-memory polymers, has enabled the creation of multi-modal, dynamic, and physically unclonable security features [22, 24–27]. Additionally, process-based authentication methods, such as the deliberate modulation of printing parameters, have been explored to enhance traceability and supply chain integrity [28, 29].

VPP stands at the forefront of anti-counterfeiting efforts in AM due to its high resolution, material versatility, and ability to embed complex security features. A recent study demonstrated the use of photoisomerisable fluorophores embedded within a polymer matrix during the VPP process. This method enabled multi-colour fluorescent patterns that were indecipherable with conventional inks and could only be revealed under specific lighting conditions [30]. Hydrogel arrays fabricated via VPP have encoded information sequentially revealed through heat-induced phase transitions or UV/temperature-triggered multi-colour fluorescence; this enables multi-level encryption where information is only accessible under specific environmental conditions [31].

Despite these advances in embedding security features within AM products, many methods remain susceptible to reverse-engineering through optical or power side-channel attacks [12]. Some advanced materials used for anti-counterfeiting purposes, such as fluorescent inks or phase-change materials, may degrade under environmental stress, thereby reducing their long-term effectiveness [27, 32]. Furthermore, many high-security features require complex post-processing or specialised materials and equipment, which invariably increases costs and limits mass-market scalability. While AI-enabled authentication systems offer improved reliability, they may introduce new vulnerabilities if adversarial machine learning attacks are considered [33, 34].

This study aims to develop a cost-effective and user-friendly method for embedding physically unclonable functions (PUFs) capable of withstanding a certain degree of environmental stress. These PUFs are created by exploiting Brownian motion to randomly embed micron-level taggants during the VPP process. This inherent randomness ensures that each resultant pattern is uniquely distinct and demonstrates substantial spatial complexity and resistance to physical replication, thereby providing robust, hardware-level security. PUFs have been widely proven to be a highly effective and leading anti-counterfeiting strategy [33, 35, 36].

The deployment of taggants is a widespread protective measure in the forensics field, allowing objects to be reliably identified, tracked, and traced [37]. Taggants are typically introduced into components at a dopant level of 5% or less, making them an affordable solution [38, 39]. However, the primary disadvantage of some basic taggants is that they are relatively easy to detect and replicate if the implementation is too simple or obvious [40]. The wide availability and low cost of taggant materials makes it easier to be duplicated.

To address this vulnerability, this study aims to develop a robust “double-lock” system. The first lock exists physically, while the second exists digitally, generating a security feature that is highly resistant to physical replication, but straightforward to authenticate. This framework could mitigate the challenges associated with identifying counterfeit goods, thereby preventing reputational and financial damage to manufacturing firms. Specifically, the spatial distribution of taggants within the printed components, alongside their subsequent effect on the part’s mechanical and thermal properties, are investigated.

The remainder of this article is organised as follows: Sect.  2 details the materials selection, while Sect.  3 describes the methods on taggant integration and detection. Section  4 presents the results and discussion, encompassing the optical taggant verification, the XCT spatial coordinate hashing, and the macro-scale mechanical and thermal validation of the parts. Finally, Sect.  5 outlines the conclusions, technical limitations, and future avenues for practical supply chain deployment. To ensure terminological precision, this study defines the overarching security framework as the “double-lock” system. Within this framework, lock 1 relies on the physical existence of the taggants; while lock 2 relies on both the “taggant code” - the physical, optically verifiable colour sequence of the embedded Microtaggants^®^, and the “spatial hash” - the digital cryptographic string mathematically derived from the taggants’ 3D XCT coordinates.

**Fig. 1:**
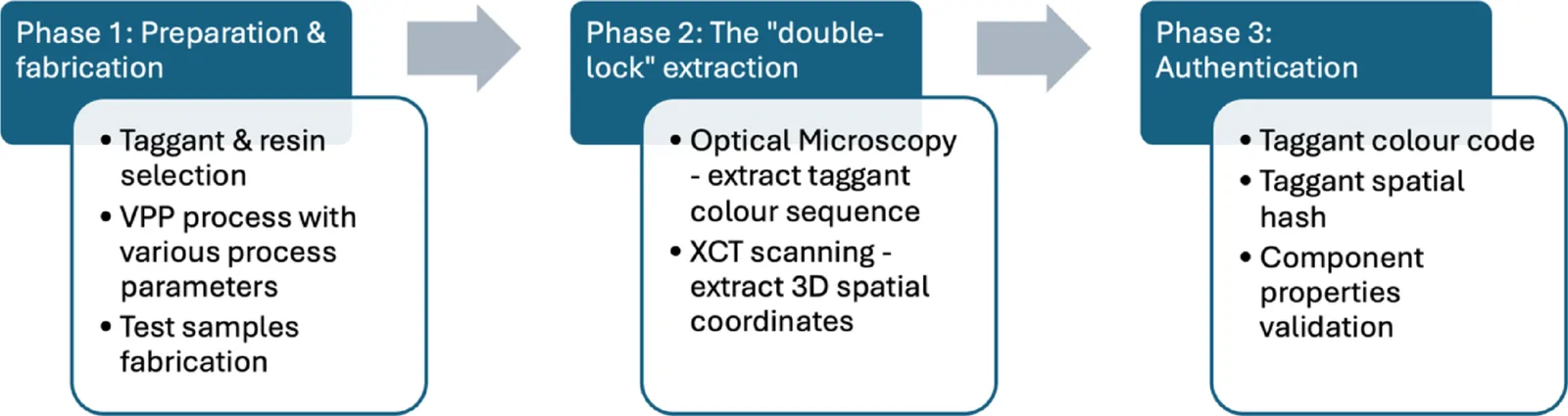
Study workflow

Figure 1 illustrates the overarching methodological workflow employed in this study, detailing the progression from material preparation through to the dual-lock authentication.

## Materials

### Taggant

Given that the primary objective of this research is to establish a practicable methodology for embedding a physical “hash” within the VPP process to safeguard the AM supply chain, a commercially available forensic taggant was selected. Existing forensic taggants include physical, spectroscopic, chemical, and DNA-based variants, and each possessing distinct analytical methods, applications, advantages, and limitations [37]. For this study, Microtaggant^®^ (Microtrace LLC, USA), a physical taggant incorporating multiple tiers of security within each individual particle, was chosen.

**Fig. 2:**
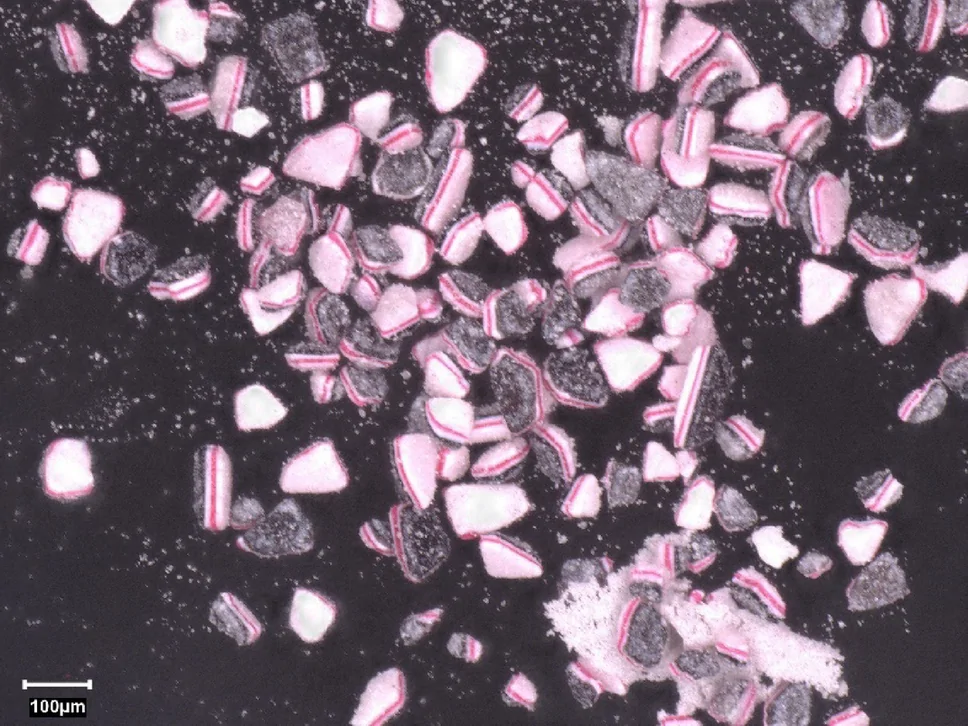
Microtaggant^®^ shows a colour layer sequence under optical microscopy

As a physical taggant, its uniqueness is derived from its specific sizing, appearance, or structural arrangement [41]. Such encoding mechanisms are frequently classified as “graphical”, given that detection and analysis are predominantly achieved via visual inspection [42]. The Microtaggant^®^ particles comprise a specific colour layer sequence (shown in Fig. 2), wherein the sequence can be translated into a unique numeric code (Table 1). This code can subsequently be registered to a specific owner within an electronic database [43].

**Table 1:** Microtaggant^®^’s colour coding values suggested by Microtrace, but can be user-defined

Coding value	Colour	Coding value	Colour
0	Black	5	Green
1	Brown	6	Blue
2	Red	7	Violet
3	Orange	8	Grey
4	Yellow	9	White

**Fig. 3:**
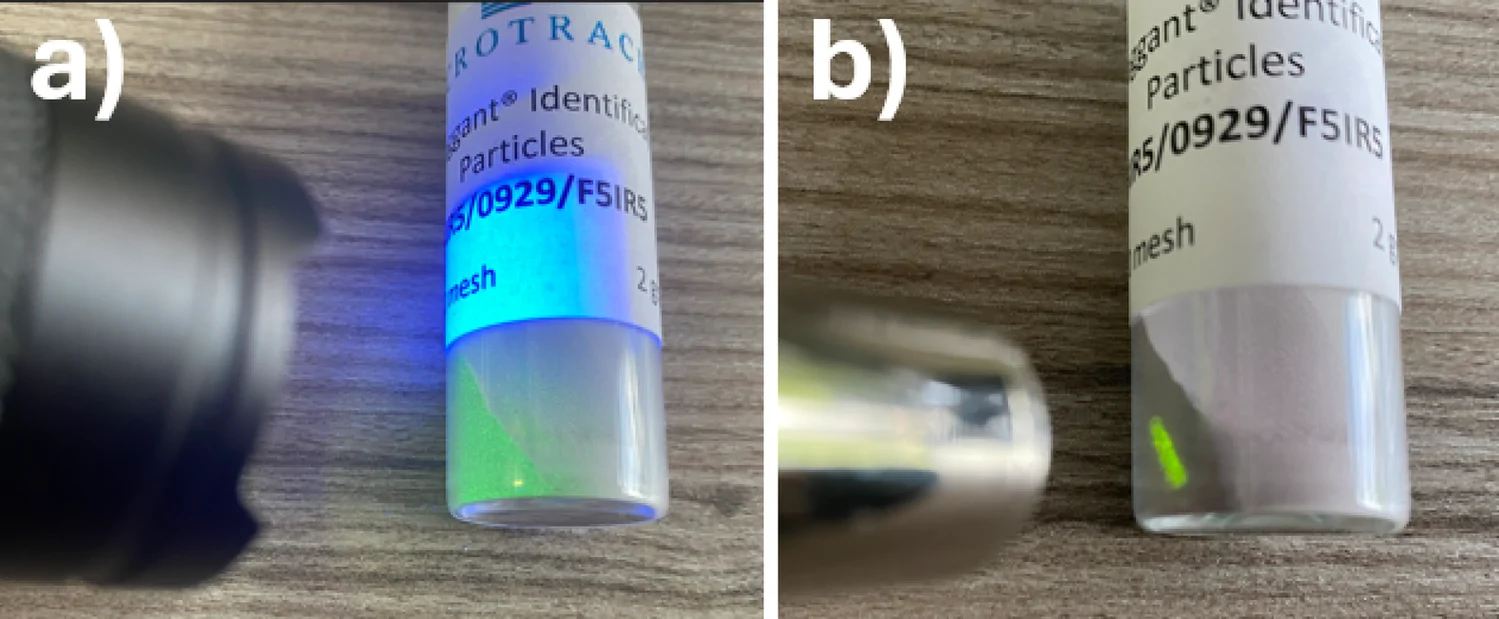
Afterglow features shown by Microtaggant^®^, **a** under 365 nm UV light, **b** under 980 nm laser pen

Furthermore, the Microtaggant^®^ is engineered with infrared (IR), ultraviolet (UV), and afterglow properties (shown in Fig. 3). This multi-modal functionality enables investigators to perform rapid, non-destructive field testing using handheld detectors. Crucially for this application, the Microtaggant^®^ remains stable at temperatures up to 250 °C and can withstand short-term exposure up to 350 °C, a characteristic conventionally leveraged in explosives detection [37].

Microtrace supplies the Microtaggant^®^ in a broad range of particle sizes, spanning from 20 to 1,200 μm. In consideration of the specific VPP layer thicknesses evaluated in this study (25–100 μm), a particle size range of 38 to 75 μm was acquired. This specific size distribution yields approximately two million particles per gram of material.

### Resin

A triacrylate-based amorphous resin, HighTemp DL400 (PhotoCentric Ltd, UK) was selected as the host matrix for this study. In its uncured state, it presents as a transparent amber liquid; upon full curing, it exhibits a high heat deflection temperature (HDT) of 230 °C. The inherent transparency of the cured polymer is highly advantageous, as it permits the embedded Microtaggant^®^ particles to be readily detected via optical microscopy without requiring destructive sectioning. Additionally, the high HDT ensures that the cured components can undergo mechanical testing at elevated temperatures, closely approaching the thermal stability limits of the embedded taggants themselves.

## Experiment methods

### Experimental plan overview

The experimental methodology is structured around three primary phases. First, resin samples are fabricated using a VPP printer across varying process parameters, specifically distinct layer thicknesses. During this phase, the taggants are randomly embedded within the parts to establish robust physically unclonable functions (PUFs). Second, the embedded taggants are detected and quantified using a combination of optical methods and X-ray computed tomography (XCT). The data acquired during this step forms the foundation of the novel double-lock security architecture; the inherently random distribution of the physical taggants serves as the primary physical hash, while the extracted spatial coordinates and volumetric data are utilised to generate a corresponding encrypted digital hash. Finally, the fabricated components undergo comprehensive thermal and mechanical testing to ensure that the integration of these high-security features does not compromise the structural integrity or performance of the final part.

### 3D printing

#### Resin preparation

Prior to printing, the HighTemp DL400 resin was heated in a gravity convection oven (Fisher Scientific Ltd, UK) at 60 °C for 5 h. This pre-heating step was necessary to dissolve any polymer crystallisation that can occur at or below ambient room temperature. Once the resin was fully liquefied, the Microtaggant^®^ particles were introduced, and the mixture was dynamically stirred at 60 °C and 500 rpm for 20 min using an AM4 Heating Magnetic Stirrer (VELP Scientifica Srl, Italy). The taggant loading concentration was maintained at 0.01 g per 100 ml of resin, aligning with the supplier’s recommendations. This ratio yields an estimated dispersion of 20,000 particles per 100 ml of liquid matrix.

#### Sample fabrication

Test specimens were fabricated using a Liquid Crystal Nano (Photocentric Ltd, UK) VPP 3D printer, which operates via bottom-up mask projection stereolithography (MPSL) utilising a liquid crystal display (LCD) mask with 4 k resolution. To evaluate the influence of layer thickness on both the quantity of embedded taggants and overall part quality, three distinct layer thicknesses were investigated (25, 50, and 100 μm).

Specific process parameters were tailored for each layer thickness to ensure thorough curing while preventing over-curing and mitigating excessive print times (summarised in Table 2). The energy density per unit power was calculated using Eq. 1. Because MPSL exposes the entire planar area simultaneously at a fixed power, energy density is calculated linearly along the build direction (J/mm), rather than volumetrically as is standard in scanning-based processes.

1 $$\:Energy\:Density\:=\:\frac{1W\:\times\:\:Exposure\:Time}{Layer\:Thickness}$$

**Table 2:** Printing process parameters for each layer thickness

Layer thickness (µm)	Exposure time for each layer (ms)	Energy density per unit power (J/mm)
100	2,500	25
50	1,500	30
25	1,000	40

The printing parameters provided in Table 2 reflect the supplier’s standardised settings. The non-monotonic relationship between energy density and layer thickness is an optimised feature to prevent over-exposure, and artificially scaling the energy density to maintain a linear trend would result in light bleed and dimensional inaccuracies. Apart from the specific parameters outlined above, the HighTemp DL400 resin was processed using standard manufacturer recommended printing profiles. Crucially, the stochastic generation of the PUF relies on inherent fluidic chaos during the recoating cycle, rather than on highly controlled kinematic variables.

Following the fabrication process, all printed samples were subjected to a 10 minute solvent wash in Tripropylene glycol monomethyl ether (TPM), succeeded by a 2 minute rinse under tap water. The specimens were subsequently detached from the build platform, wiped clean, and allowed to air-dry. Final post-curing was conducted in a 400 nm ultraviolet-visible (UV-Vis) oven (Cure L2, Photocentric Ltd, UK), where the samples were heated at 60 °C for 2 h to achieve full mechanical properties.

#### Test specimen specifications

Tensile test specimens conforming to the ASTM D638 Type V standard [44] were fabricated utilising the process parameters outlined in Table 2. For each of the three evaluated layer thicknesses, a total of 40 specimens were produced. To accurately isolate the mechanical impact of the additives, these were divided equally: 20 specimens per layer thickness contained embedded taggants, while the remaining 20 were printed using neat resin to serve as control samples. All tensile specimens were printed in an upright orientation, with their longitudinal axis aligned parallel to the Z-build direction and were adhered directly to the build platform without the use of support structures.

To accommodate static tensile testing at elevated temperatures, an additional cohort of specimens was manufactured exclusively using the 100 μm layer thickness parameter set. For each designated test temperature, 12 specimens were produced (comprising 6 tagged samples and 6 neat resin controls). These were identically oriented in the upright position and printed directly onto the substrate.

Furthermore, to facilitate thermal analysis, cylindrical discs measuring 5 mm in diameter and 1.5 mm in thickness (Ø5 × 1.5 mm) were fabricated. A total of 36 discs (18 embedded with taggants and 18 neat resin controls) were printed concurrently within the same print job as the 100 μm layer thickness tensile specimens.

### Taggants detection

#### Visual detection

To verify the successful embedment of the taggants within the fabricated components, initial non-destructive inspections were conducted utilising a 365 nm portable UV light and a 980 nm infrared laser pen (Microtrace LLC, USA). Subsequently, a VHX7000 digital optical microscope (Keyence Ltd, UK) was employed to optically resolve the colour layer sequences of individual taggant particles, allowing the corresponding numeric values to be obtained and verified.

To ensure precise detection and authentication, an optical verification protocol was established. First, the global presence of the taggants was confirmed macroscopically via handheld UV (365 nm) and IR (980 nm) excitation. Subsequently, specimens were examined via optical microscopy, where the focal plane was adjusted precisely to the sub-surface layers of the cured resin. This allowed the distinct, multi-layered colour sequence of the isolated Microtaggants^®^ to be visually resolved and manually verified against the taggant code.

#### X-ray computed tomography (XCT)

X-ray computed tomography (XCT) was utilised to extract and characterise the size, number and position of taggants within the samples, a technique perfectly suited to evaluating and quantifying materials across engineering sectors [45–48]. A total of 9 different tensile test samples were scanned using XCT, three at each of the layer thickness conditions that can be observed in Tables 2 and 100 μm, 50 μm, and 25 μm. The central-most section of each was scanned on a Tescan Unitom XL system (Tescan-Orsay, Brno), at the Centre for Imaging, Metrology, and Additive Technologies (CiMAT). All samples were scanned at the same settings using the proprietary scanning software, Acquila (Tescan-Orsay, Brno), using a polychromatic source with a tungsten target. A beam voltage of 80 kV was used at a power of 15 W. A total of 2279 projections were acquired with a Source-Detector Distance (SDD) of 1250 mm, an exposure time of 0.51 s, 2 frame averages and at a voxel size of 3 μm. The datasets for all nine samples were reconstructed in Panthera (Tescan-Orsay, Brno) using a standard FDK algorithm [49], producing a set of tiff image stacks for each sample representing a 5 mm section of the centre of each sample.

These tiff stacks were then analysed in Avizo 2021.2 (Thermo-Fisher Scientific, Waltham) to extract a few properties for each sample, namely: (1) the volume fraction of taggants; (2) the number of taggants; (3) the volume and position of individual taggants.

For all samples, individual taggants were segmented in Avizo using an Interactive Threshold module, with variable settings applied as detailed in Table 3. These variable thresholds were strictly necessary to compensate for inherent XCT scanning artefacts, such as minor beam hardening and scan-to-scan background noise fluctuations. Crucially, the proposed PUF architecture relies on the extraction of taggant centroids (spatial coordinates) rather than precise volumetric measurements. Because minor variations in segmentation thresholds primarily affect the calculated surface boundaries of the particles rather than their central coordinates, the topological integrity of the spatial hash and the subsequent randomness analysis remain robust against potential operator bias. Following this initial thresholding, residual noise was removed using an Opening module with a 2px cube structuring element and an 18px neighbourhood. The surrounding resin was subsequently segmented using the Wand Tool (settings also provided in Table 3), accompanied by a sequence of dilation and erosion to further eliminate noise and fill internal voids. This comprehensive protocol produced the final label fields for formal analysis.

Volume fractions were calculated as a percentile of each fraction to the total volume of both fractions. Volume and position characteristics were extracted using the Label Analysis module, extracting Volume3d, BaryCenterX, BaryCenterY, and BaryCenterZ.

**Table 3:** Thresholding parameters for fractions in all samples in avizo

Sample	Taggant threshold	Resin threshold
100 μm #1	21,399–65,535	9773–21,398
100 μm #2	15,158–65,535	5479–15,159
100 μm #3	18,724–65,535	7473–18,723
50 μm #1	31,653–65,535	17,533–31,652
50 μm #2	38,340–65,535	18,971–38,339
50 μm #3	22,291–65,535	9198–22,290
25 μm #1	34,328–65,535	20,408–34,327
25 μm #2	31,207–65,535	18,971–31,206
25 μm #3	23,182–65,535	11,497–23,181

### Property evaluation

#### Mechanical testing

Room temperature tensile specimens were tested to failure under static tension at a constant crosshead speed of 1 mm/min. These tests were conducted using an Instron 3367 test system (Instron UK Ltd, UK) equipped with a 30 kN static load cell and an Instron 2630-102 clip-on extensometer.

Elevated temperature tensile testing was similarly performed at 1 mm/min, utilising an Instron 5985 test system (Instron UK Ltd, UK) fitted with a 10 kN static load cell, a dedicated environmental chamber, and an Instron 2663-821 advanced video extensometer. Testing was carried out at discrete temperatures of 23, 60, 100, 140, 180, and 220 °C. Once the environmental chamber reached the designated target temperature, the specimens were subjected to an additional 5 minute isothermal soaking period to ensure uniform thermal distribution prior to test initiation. Concurrently, six disc samples (comprising three embedded with taggants and three neat resin controls) were placed inside the environmental chamber during the tensile testing process. This ensured the discs acquired a thermal history identical to that of the tensile specimens for subsequent comparative analysis.

#### Thermal analysis

Differential Scanning Calorimetry (DSC) was performed on the fabricated disc samples using a DSC 1 thermal analysis system (Mettler Toledo UK, UK). All samples were encapsulated within aluminium pans with lids and evaluated under a steady nitrogen atmosphere. The programmed thermal cycle consisted of an initial heating phase from 25 to 250 °C at a ramp rate of 20 °C/min. Upon reaching 250 °C, the samples were held isothermally for 5 min, followed by a controlled cooling phase back to 25 °C at a rate of 20 °C/min. This identical thermal protocol was applied to both the taggant-embedded samples and the neat resin controls.

## Results and discussion

### Taggant detection and characterisation

#### Visual verification of taggant embedment

In the tensile test specimens fabricated using 25, 50, and 100 μm layer thicknesses, taggants were successfully detected across all samples under both 980 nm infrared and 365 nm UV light exposure. These observations demonstrate that the Microtaggant^®^ particles can be reliably embedded within the resin matrix via the vat photo-polymerisation (VPP) process, even when the specified printing layer thickness is nominally smaller than the taggant particle size (38–75 μm).

**Fig. 4:**
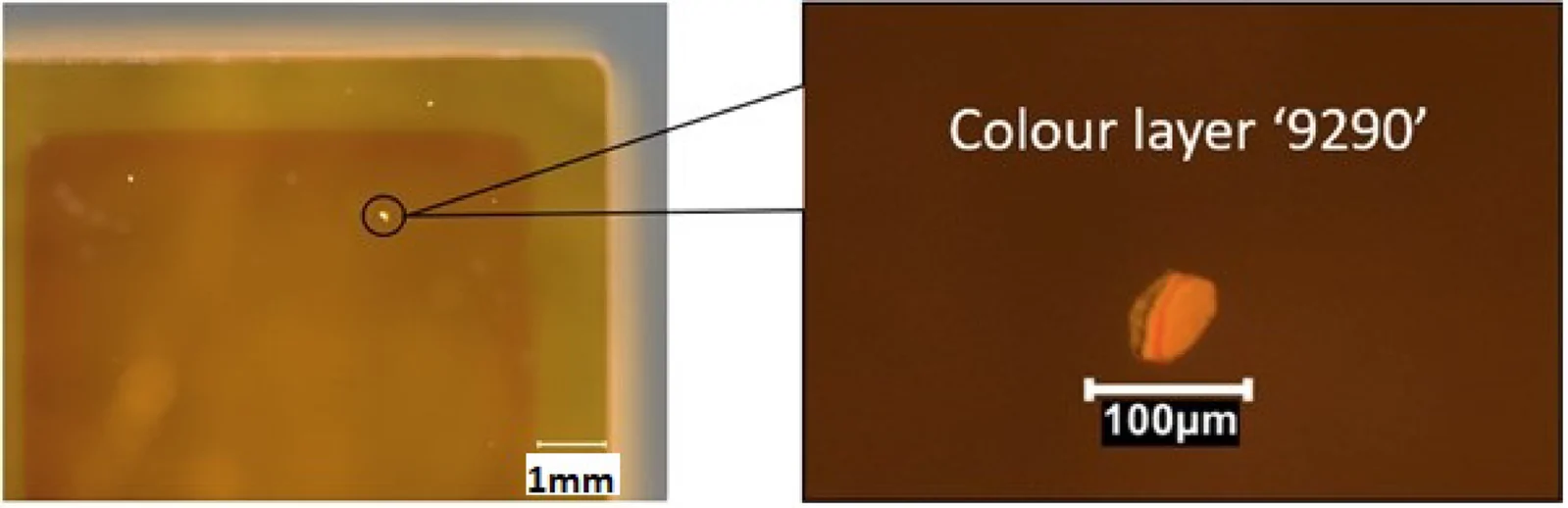
Embedded taggant detected by Keyence VHX700 optical microscope

During printing at a 25 μm layer thickness, it is posited that the taggants are transported into the gap between the aluminium build plate and the polymer vat sealing film during the resin filling stage, a point in the cycle when the gap significantly exceeds 25 μm. As the build platform descends and the curing gap closes to the final 25 μm thickness, the particles are subsequently pressed into the compliant vat film or the previously cured resin layers. Figure 4 demonstrates that even after the AM process, embedded taggants can be successfully isolated, and their complex, multi-layered colour codes (which serve as the primary sequence of the digital hash) can be resolved and validated against a master database.

#### Volume fraction of taggants

Determining the exact volume fraction of the embedded Microtaggants^®^ is a critical step in validating the proposed security architecture. The internal loading ratio must strike a precise physical balance: it must be sufficiently high to generate a complex and unclonable 3D spatial hash, yet remain low enough to prevent particle agglomeration. Furthermore, an excessive volume fraction risks obstructing the UV photon penetration during the VPP process, which would compromise both the resin curing kinetics and the mechanical integrity of the components. Therefore, XCT volumetric analysis was deployed to verify that the optimal loading was achieved.

The volume fractions of the embedded taggants for each sample are detailed in Table 4. Generally, the volume fraction remains exceedingly low across all samples but exhibits a clear positive correlation with increasing layer thickness.

**Table 4:** Volume fraction of taggants in each sample

Sample layer thickness	Volume fraction of taggants (%)	Volume fraction of resin (%)
25 μm	0.0004 ± 0.0002	99.9996 ± 0.0002
50 μm	0.0008 ± 0.0004	99.9992 ± 0.0004
100 μm	0.0048 ± 0.0008	99.9952 ± 0.0008

#### Dimensions and quantity of taggants

The number of taggants and their volumetric data can be found in Table 5. Overall, the number of taggants appears to scale with the layer thickness, with coarser thicknesses resulting in the retainment of more taggants. The size of taggants in each sample appears to be quite variable however, with a wide variety of taggant sizes being present in each sample with a large standard deviation for each. This appears to imply overall that coarser layer thicknesses result in greater retention of taggants, but the size of those taggants is also variable.

**Table 5:** Properties of taggants in samples

Sample	No. of taggants	Average volume of taggants (mm^3^) x10^− 5^
100 μm #1	18	9 ± 7
100 μm #2	20	15 ± 10
100 μm #3	27	9 ± 6
50 μm #1	6	4 ± 3
50 μm #2	5	5 ± 3
50 μm #3	12	6 ± 4
25 μm #1	1	10.1
25 μm #2	7	2 ± 5
25 μm #3	4	10 ± 7

#### Spatial Distribution and Randomness Analysis

**Fig. 5:**
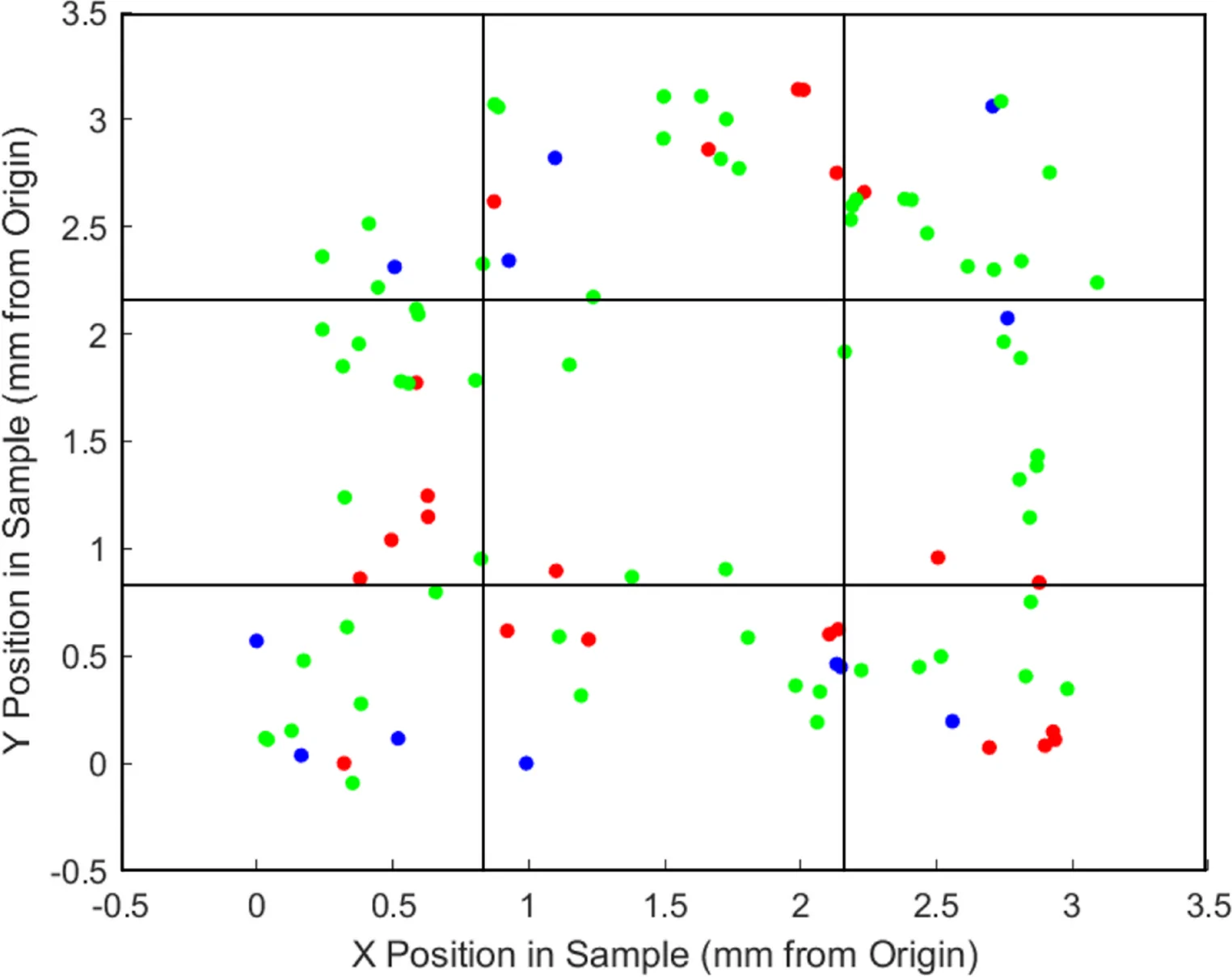
XY positions of taggants along Z-axis of each sample: blue 25 μm, red 50 μm, green 100 μm layer samples

**Fig. 6:**
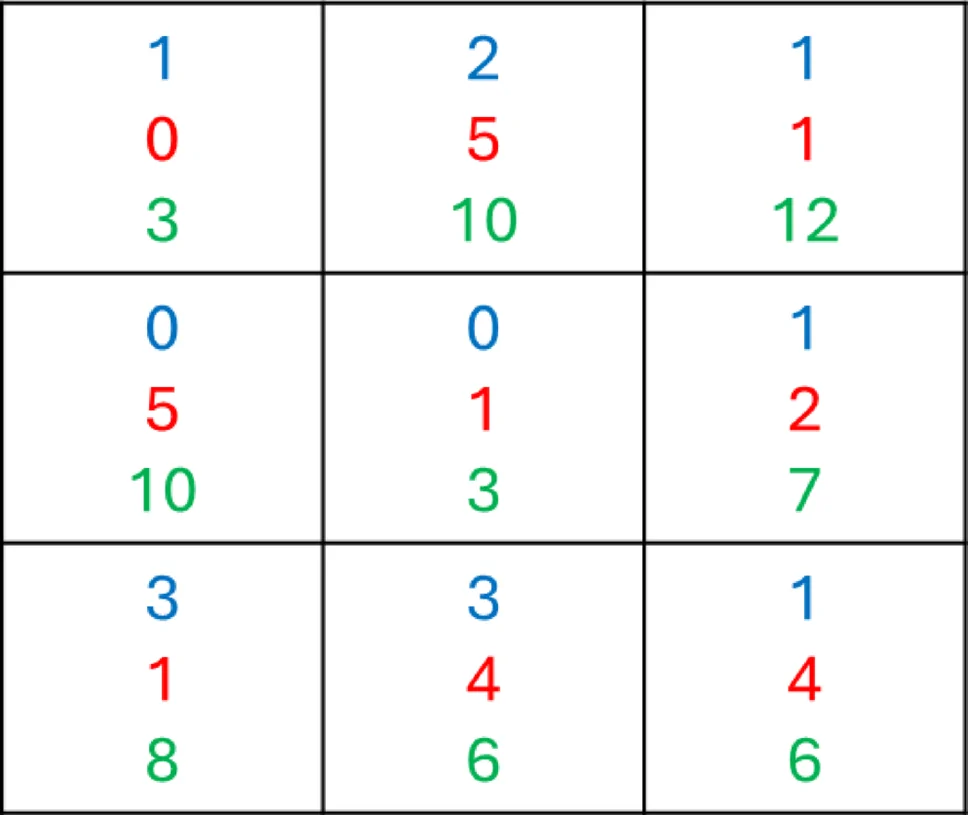
The count of taggants in each cell: blue 25 μm, red 50 μm, green 100 μm layer samples

The spatial coordinates of the taggants within each sample were plotted to assess their dispersion (shown in Fig. 5). To statistically evaluate the uniformity of the taggant distribution, a quadrat analysis was conducted using a 3 × 3 grid; the frequency of observations within each resulting cell was recorded (shown in Fig. 6). Initial visual inspection suggests a spatial bias, with taggants appearing to cluster towards the corners of the samples and relatively fewer locating in the central regions. This phenomenon also appears dependent on layer thickness, as coarser layers exhibit a comparatively higher concentration of centrally located taggants.

To rigorously test the observed quadrat distributions for Complete Spatial Randomness (CSR), each distribution was modelled against a uniform Poisson distribution, and a c^2^ goodness-of-fit test was performed. The test was performed using a 5 × 5 square quadrant. The analysis was executed using the “quadrat.test” function within the *spatstat* spatial point pattern analysis package in R (The R Foundation). To ensure statistical rigour, the evaluation framework was defined as follows:

- Null Hypothesis ($\:{H}_{0}$): The spatial distribution of the taggants follows a completely random pattern (consistent with a uniform Poisson distribution).
- Alternative Hypothesis ($\:{H}_{1}$): The spatial distribution of the taggants does not follow a random pattern (exhibiting spatial bias, clustering, or regularity).

The significance threshold was established at *α*= 0.05. Under this framework, a resulting p-value ≥ 0.05 indicates a failure to reject the null hypothesis, meaning the taggant distribution is considered statistically random. Conversely, a p-value < 0.05 dictates the rejection of $\:{H}_{0}$, indicating a non-random distribution.

The results of this analysis are presented in Table 6. Applying the established criteria reveals that the 25 μm and 50 μm sample distributions generally exhibit complete spatial randomness (p-value > 0.05, fail to reject $\:{H}_{0}$). Conversely, all three samples fabricated at the 100 μm layer thickness yielded p-values < 0.05, dictating the rejection of the null hypothesis and indicating a clustered, non-uniform distribution. While the 25 μm layers achieve mathematical uniformity, the 100 μm layer thickness induces localised micro-clustering of the taggants. In the context of PUF security, this non-uniform clustering is highly advantageous; the unpredictable formation of dense taggant clusters and spatial voids creates a more complex, overlapping physical topology. This drastically increases the structural entropy of the security feature, rendering the 100 μm prints significantly more resistant to physical replication than a perfectly distributed grid.

**Table 6:** Results of χ^2^ test for randomness for the 25 μm, 50 μm and 100 μm distributions

Sample	Test	Sample 1	Sample 2	Sample 3
25 μm	χ^2^	25.143	21.000	24.000
	P	0.796	0.723	0.923
50 μm	χ^2^	27.333	20.000	42.167
	P	0.578	0.606	0.025
100 μm	χ^2^	47.556	47.400	48.000
	P	**0.006**	**0.006**	**0.005**

It should be noted that the physical kinetics of particle settling during the printing process inherently contribute to the stochastic nature of the taggant distribution. Because a bottom-up VPP system is utilised, gravity assists in driving the particles towards the curing interface, ensuring high embedding rates. Crucially, the unpredictable micro-dynamics of this settling process prevent the perfect reproducibility of the spatial distribution. In the context of a PUF, this lack of reproducibility is highly advantageous, as it provides the physical entropy necessary to prevent cloning and ensure each tag remains cryptographically unique.

#### The “Double-Lock” hash generation

Utilising the X-ray Computed Tomography (XCT) data, the precise physical locations of the embedded taggants can be extracted and mapped as XYZ coordinates. To account for inherent minor variations in scanner calibration or environmental noise between the manufacturer and the end-user (error tolerance), these raw coordinates are first subjected to a “coordinate binning” protocol. By rounding the coordinates to a predefined spatial tolerance (e.g., the nearest 10 μm), minor measurement discrepancies are normalised. For instance, a taggant identified with the colour code “09290” located at raw coordinates X = 3.046 mm, Y = 2.576 mm, and Z = 1.367 mm would be rounded to X = 3.05 mm, Y = 2.58 mm, and Z = 1.37 mm, and subsequently concatenated to generate a unique, part-specific raw PUF response: 09290305258137.

In a practical deployment, this concatenated string serves as a high-entropy pre-hash identifier rather than the final cryptographic key. To align with established security architectures, this raw response would be processed through a standard cryptographic hash function, such as SHA-256, generating the irreversible, fixed-length digital signature that is ultimately secured on the distributed ledger. The collision resistance of this system is robust; the vast physical entropy generated by combining millions of potential colour permutations with highly granular, randomised 3D spatial coordinates creates a highly complex physical signature. Consequently, within the scope of our tested sample cohort, no two distinct components yielded identical raw PUF responses.

**Fig. 7:**
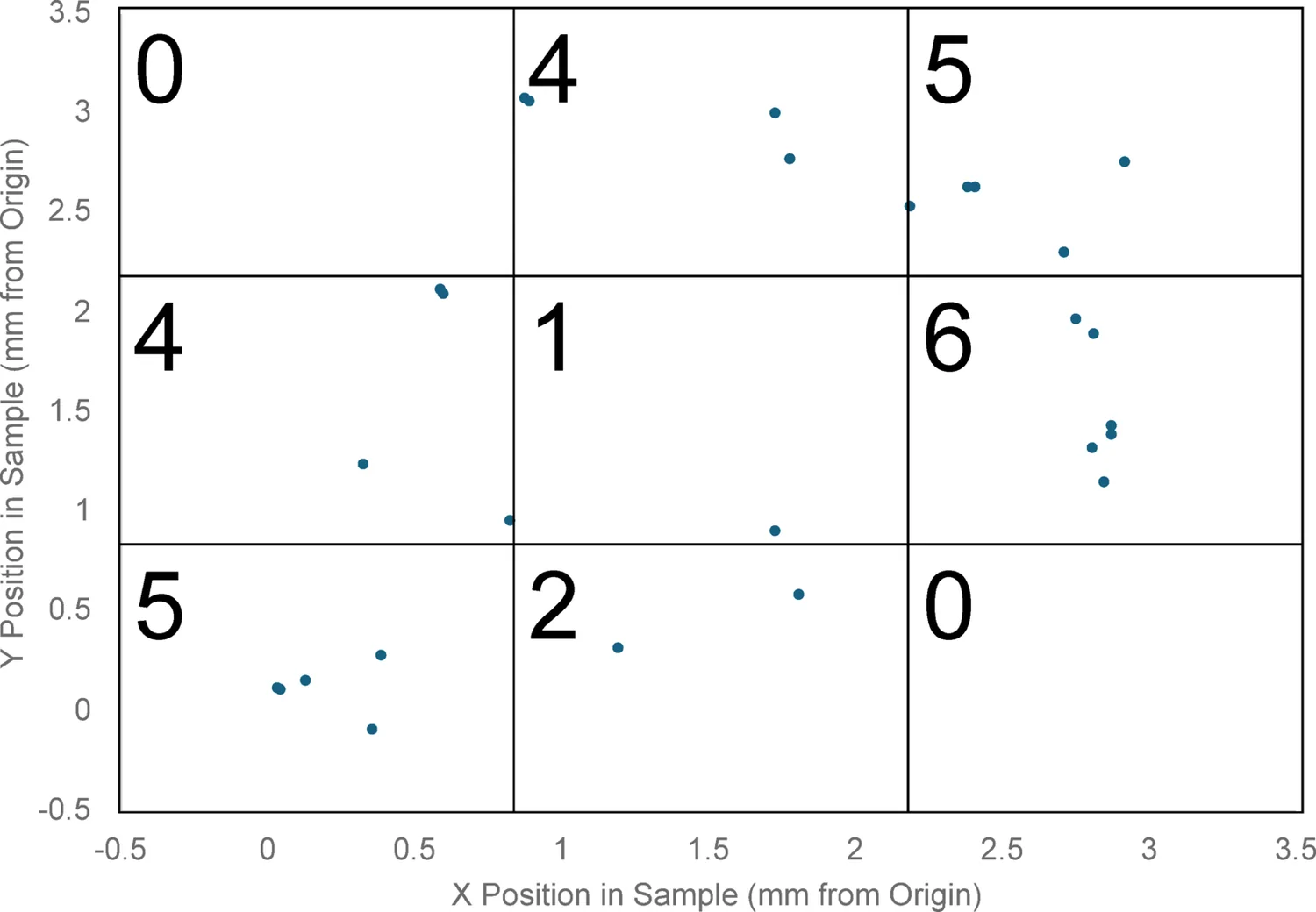
Quadrat statistics of sample 100 μm #3 taggant distribution

An alternative digital hash can be derived directly from the quadrat statistics of the spatial distribution. For example, reading the cell frequencies sequentially from the top-left to the bottom-right of a 3 × 3 grid generates a unique numeric code (e.g., 233306631), shown in Fig. 7. While this 3 × 3 grid serves as an illustrative proof-of-concept for the hashing methodology, its limited keyspace ($\:{10}^{9}$) is not collision-resistant for commercial deployment. Industrial applications would require scaling to larger grid matrices alongside the full 3D spatial coordinate authentication to ensure absolute cryptographic security.

As demonstrated by the statistical data in Table 6, utilising the spatial distribution as a supplemental digital lock is only viable for the 100 μm samples. The distributions within the 25 μm and 50 μm samples are too highly variable, primarily due to the low absolute retention of taggant particles at these finer layer thicknesses, to serve as a reliable cryptographic baseline under the current dosing methodology.

**Fig. 8:**
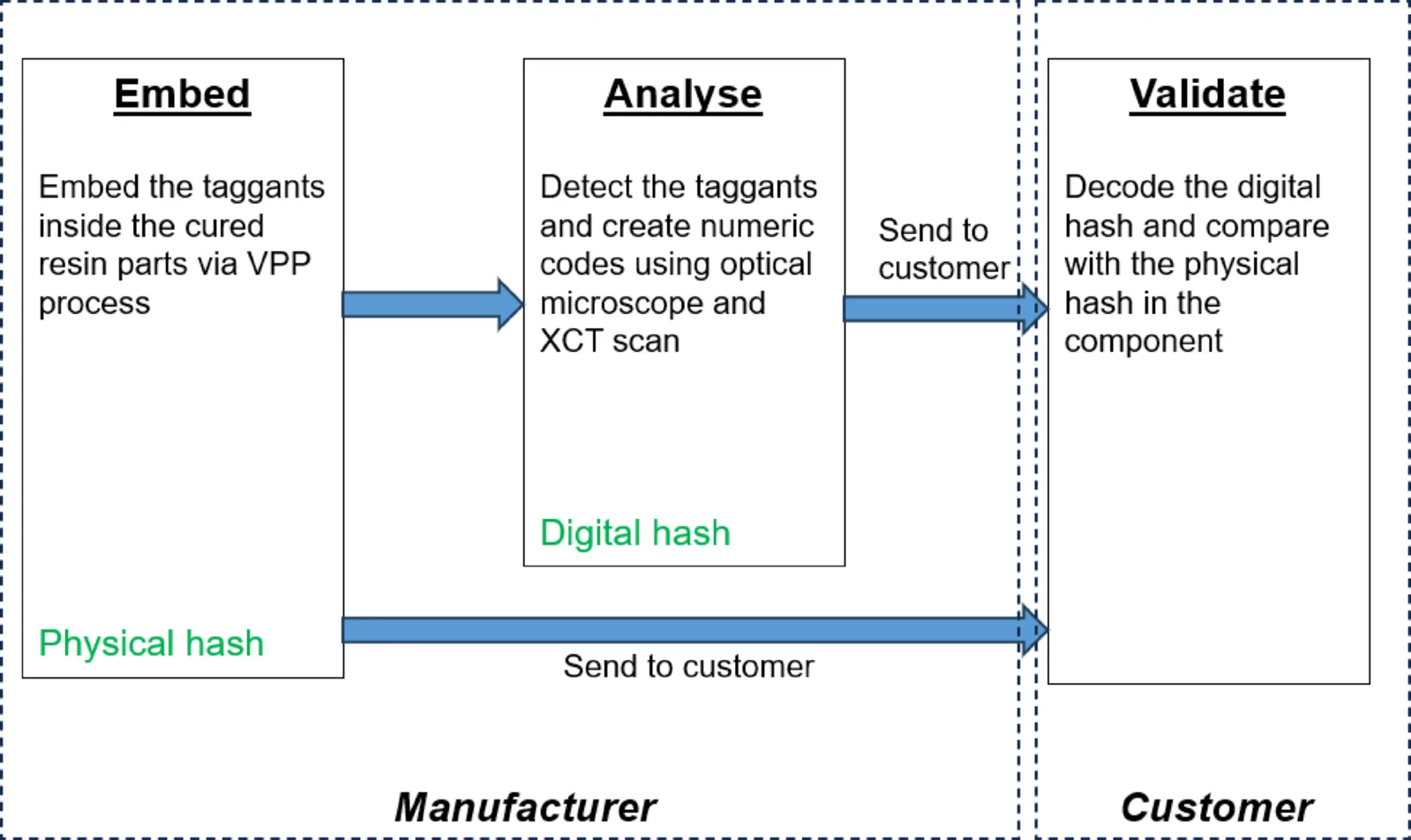
Taggants embedding and detection process

Figure 8 illustrates the taggant embedment and detection workflow into a supply chain context. At the manufacturer’s facility, post-printing, an XCT scan is performed to quantify and locate the taggants, mapping their spatial distribution to generate the secondary component of the digital hash. Subsequently, optical microscopy is utilised to extract the individual colour codes, completing the unique digital hash for each embedded particle.

The physical component and its corresponding digital hash are then transmitted to the customer via separate channels; notably, the digital hash can be securely logged onto a distributed ledger, such as a blockchain. Upon receipt, the customer initiates the verification protocol by first decoding the hash to identify the expected spatial coordinates of the taggants within the component. Visual detection methods are then employed to confirm both the precise spatial locations of the particles and the matching of their respective colour codes against the digital hash, thereby fully authenticating the part.

In instances involving complex geometric features where optical microscopy is unfeasible, or when utilising opaque resin systems, extracting the numeric value from the taggants’ colour layers presents a significant challenge. Under such circumstances, the spatial coordinates and volumetric point analysis derived from the XCT data can exclusively constitute the digital hash. Consequently, decoding and authenticating the embedded taggants would necessitate XCT scanning at the customer’s end. This exclusively XCT-based verification approach is equally applicable to the digital hash generated via quadrat statistics, requiring both the manufacturer and the end-user to perform parallel XCT evaluations.

Crucially, to ensure the verification methodology remains scan-agnostic, a singular “master taggant” is designated within the sample. The spatial coordinates of all subsequent taggants are then defined relatively, mapped against this master particle’s position. This relative coordinate framework effectively mitigates the risk of verification failure arising from the end-user employing a different scanning coordinate system or part orientation compared to the manufacturer’s original scan.

### Thermal analysis

**Fig. 9:**
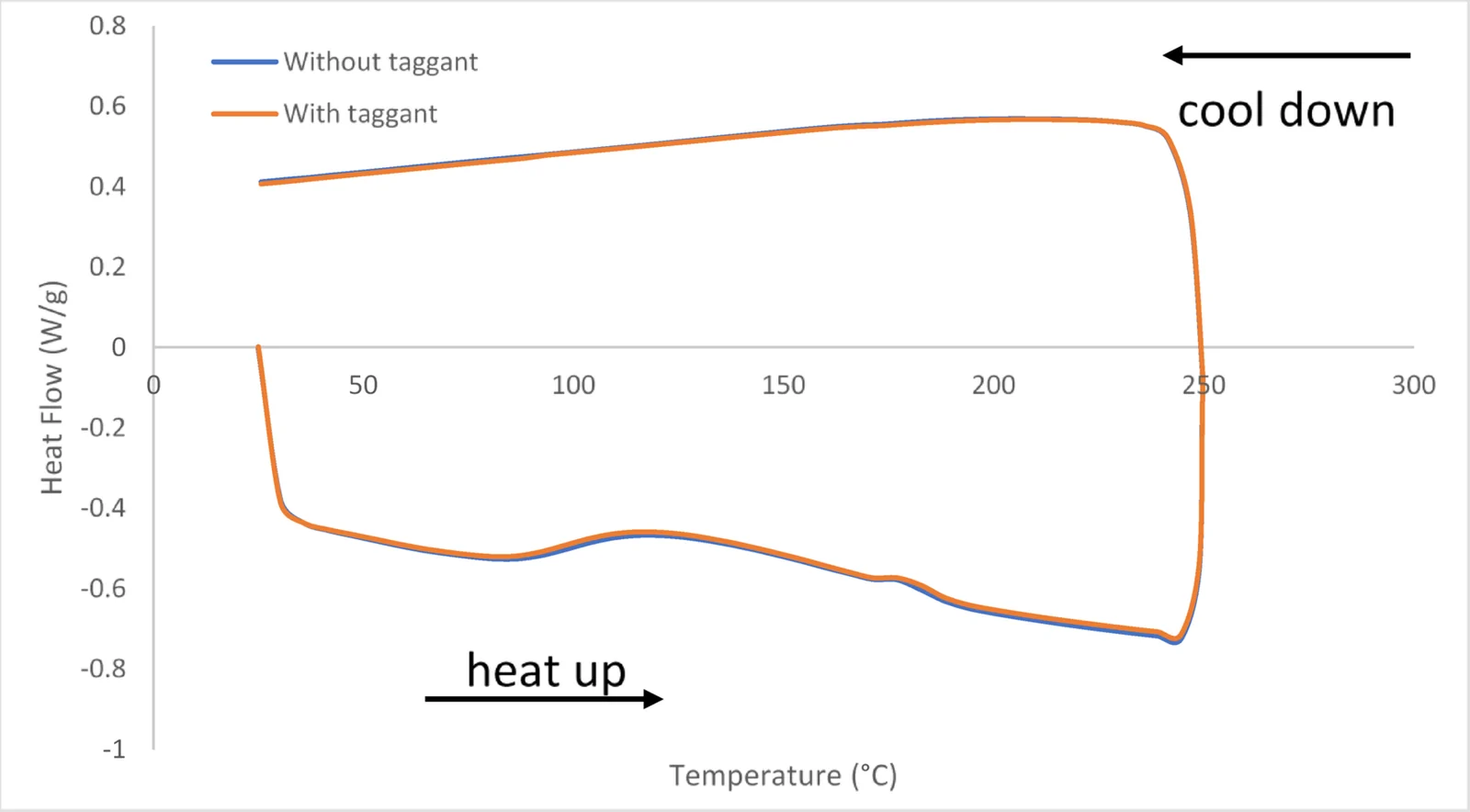
Heat flow curves of resin samples with and without taggant embedded

Figure 9 illustrates the heat flow comparison for the 100 μm layer thickness printed samples, both with and without embedded taggants. Notably, no significant difference was observed between the two conditions. Although the Microtaggant^®^ particles consist of a disparate material and possess distinct intrinsic properties, their integration did not alter the bulk thermal properties of the resin. This thermal stability is attributed to the extremely low volume fraction of the taggants within the matrix (as established in  4.1.2) and the chemical non-reactivity between the particulate matter and the surrounding photopolymer.

**Fig. 10:**
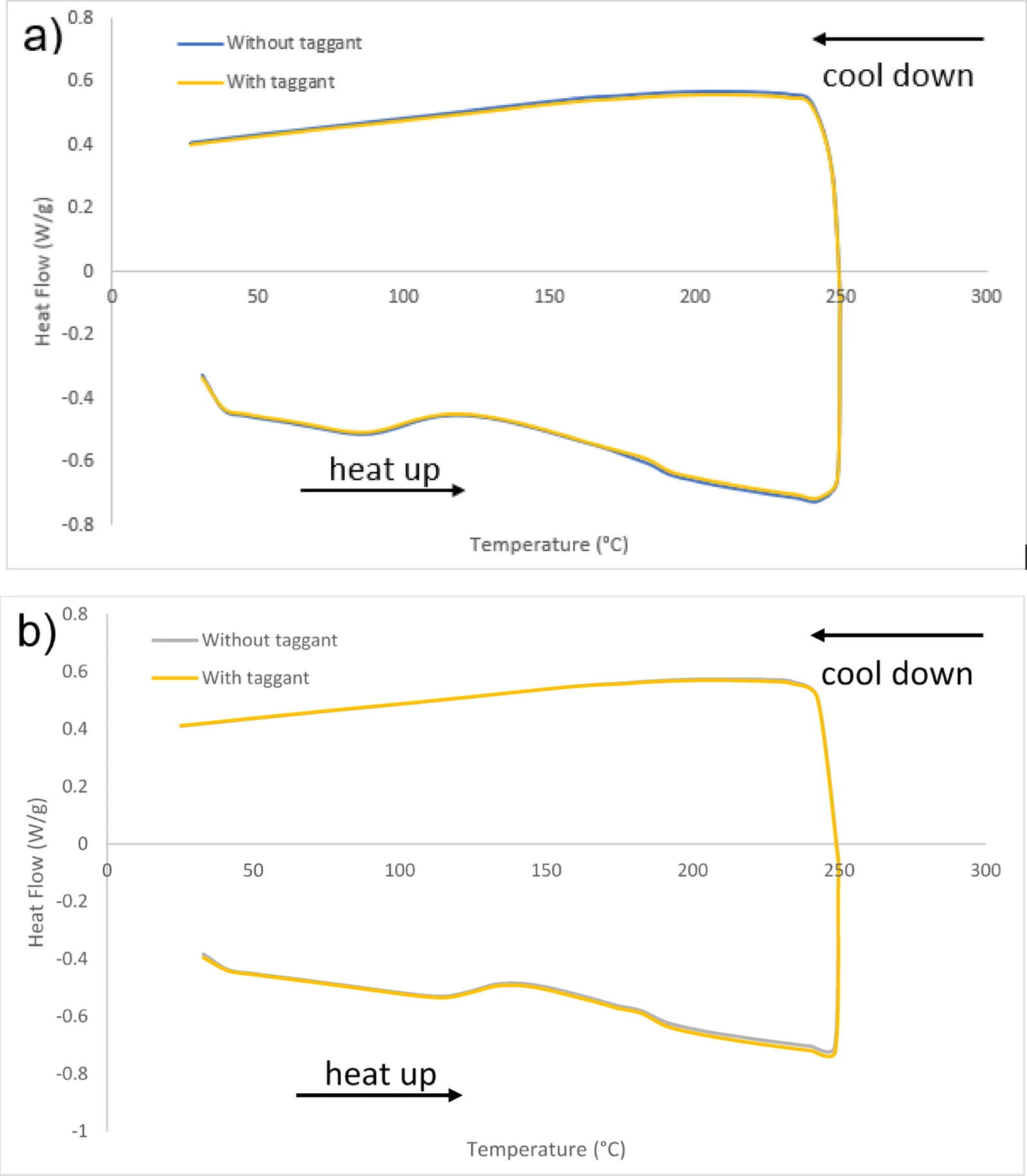
Heat flow curves of resin samples with and without taggant embedded: **a** pre-soaked at 60 °C; **b** pre-soaked at 220 °C

Given that the taggants may exhibit varying thermal conductivities, there was a theoretical potential for divergent thermal behaviour under pre-heating conditions. To investigate this, samples pre-soaked at elevated temperatures were evaluated using DSC. The heat flow profiles for samples pre-soaked at 60 °C and 220 °C, representing the lower and upper extremes of the tested elevated temperature range, are presented in Fig. 10. Consistent with the findings from the non-preheated samples, the inclusion of embedded taggants induced no discernible alterations in the thermal properties of the printed resin components.

### Static tensile testing

**Fig. 11:**
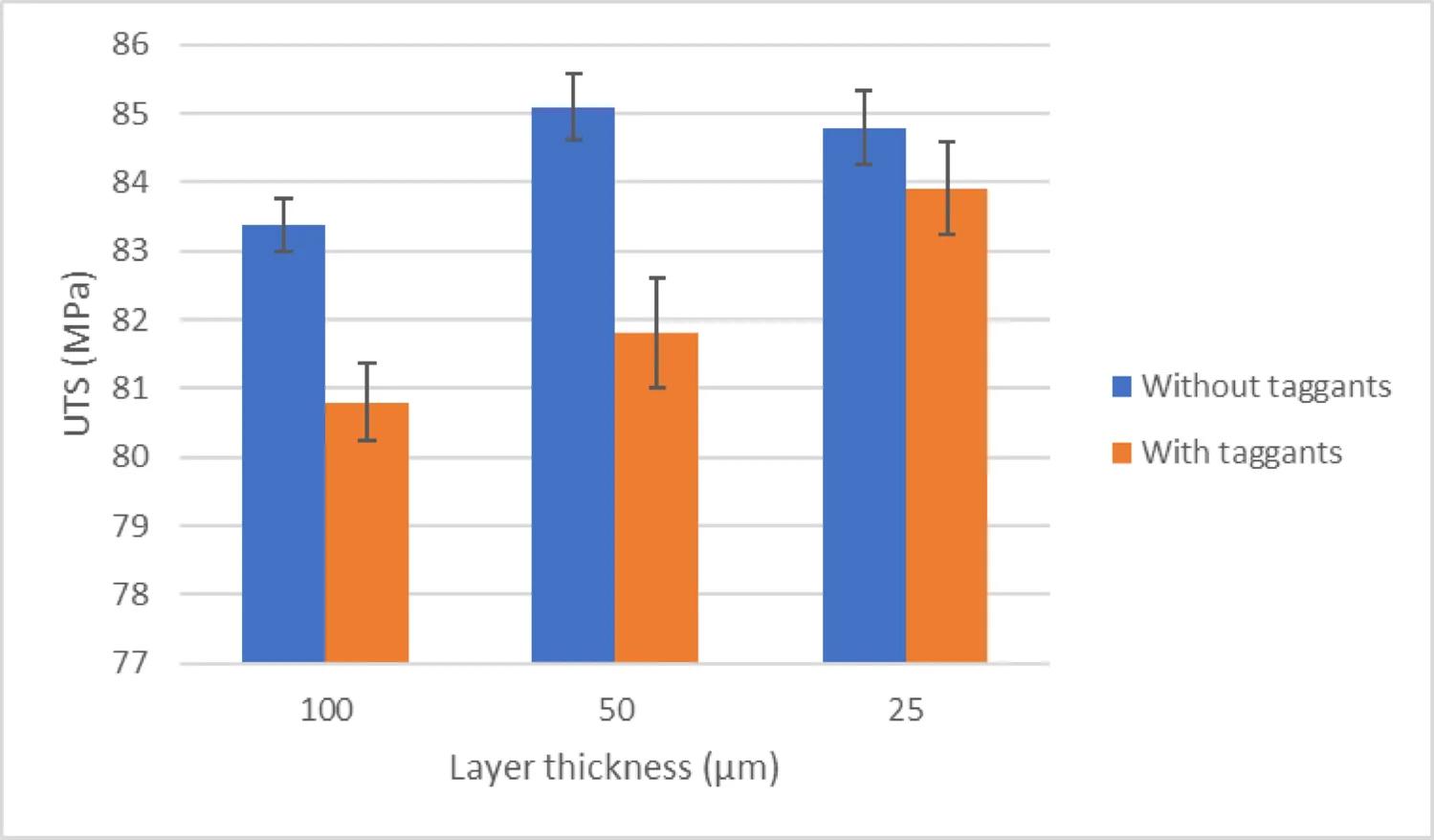
Comparison of UTS for samples printed with and without embedded taggants forroom temperature testing (error bars represent one standard deviation from the mean (*n* = 20)). Note, y-axis starts at a non-zero value.

Figure 11 presents the Ultimate Tensile Strength (UTS) values of the tensile test specimens fabricated at each layer thickness, comparing samples with and without embedded taggants at room temperature.

In the control samples devoid of embedded taggants, decreasing the layer thickness from 100 to 50 μm resulted in a measurable increase in UTS, rising from 83.4 ± 0.4 to 85.1 ± 0.5 MPa. This improvement is likely attributable to the increased energy density achieved during the curing phase [50]. Conversely, a further reduction in layer thickness to 25 μm yielded a marginal decrease in UTS (84.8 ± 0.5 MPa) compared to the 50 μm samples. Although this variation falls within the standard margin of error, it highlights a known phenomenon, that lower layer thicknesses allow the curing light to penetrate previously cured layers multiple times. This repeated exposure can induce internal residual stresses within the samples during the printing process [51], subsequently affecting overall mechanical strength.

The VPP process relies upon the liquid photopolymer undergoing a rapid chemical reaction and curing under specific light irradiation. The presence of solid taggants suspended within the liquid resin can interrupt or impair this photoinduced polymerisation [52]. Furthermore, these embedded particles may physically hinder further cross-linking during the post-curing phase. This hypothesis is supported by the tensile test results, where higher taggant densities correlated directly with diminished mechanical performance. Specifically, the 100 μm layer samples, which retained the highest volume of taggants, displayed the lowest UTS; conversely, the 25 μm layer samples retained the fewest taggants and exhibited the highest UTS among the embedded specimens. Overall, the inclusion of taggants resulted in minor UTS reductions of 3.1 ± 0.8 %, 3.9 ± 1.1 %, and 1.0 ± 1.0 % for the 100 μm, 50 μm, and 25 μm layer thicknesses, respectively, compared to their pure resin counterparts. For the 25 μm samples, the extremely low percentage of retained taggants may have mitigated the potential internal stress caused by multiple light exposures, leading to a higher UTS than that observed in the 50 μm embedded samples.

**Fig. 12:**
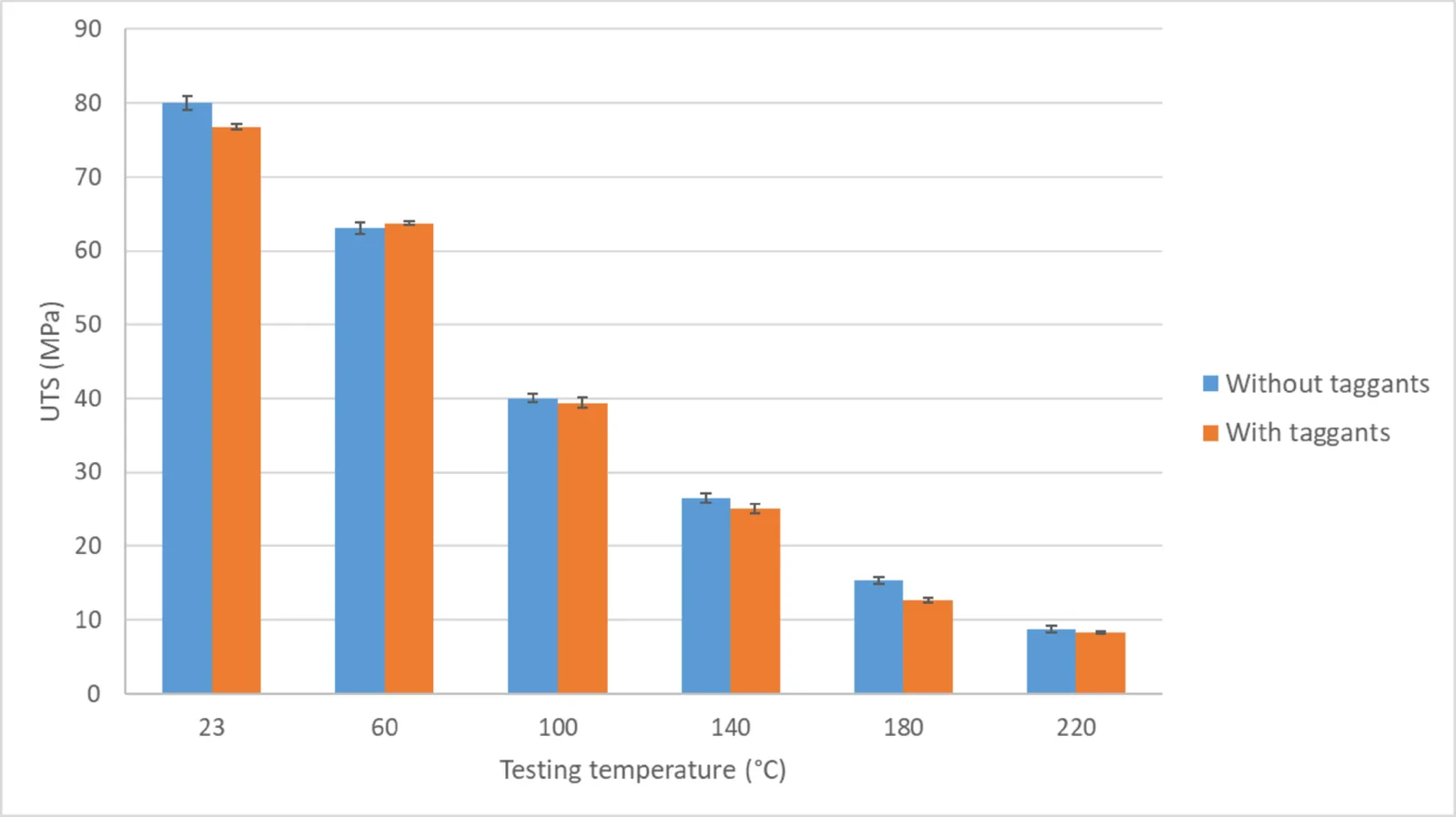
Comparison of UTS for samples printed with and without embedded taggants—elevated temperature testing (Error bars represent one standard deviation from the mean (*n* = 6))

Elevated temperature tensile testing was exclusively conducted on the 100 μm layer thickness samples, with the results detailed in Fig. 12. Crucially, the presence of the taggants did not alter the UTS beyond the standard margin of error across the elevated temperature spectrum. While relative differences in UTS of 4.0 ± 1.3 % (at 23 °C) and 17.4 ± 3.8 % (at 180 °C) were noted between the pure and embedded samples, these variations are primarily attributed to the constraints of the small sample size (*n* = 6 per test set) rather than a fundamental material degradation caused by the taggants. The apparent 17.4 % decrease in UTS observed at 180 °C must be contextualised by the overall thermal softening of the polymer matrix. Because the UTS of the pure resin drops to approximately 15.3 MPa at this elevated temperature, a standard physical deviation of merely 2.7 MPa registers as a highly inflated percentage drop. Furthermore, at 180 °C, the polymer is actively traversing its highly variable transition zone, leading to erratic yielding behaviour around the rigid embedded taggants. As observed in the 220 °C data, once the composite fully transitions into its rubbery plateau, allowing for uniform plastic flow around the taggants, the relative strength variance between the pure and embedded samples normalises.

## Conclusions

This study has demonstrated that Microtaggants^®^ can be successfully embedded into resin components via the vat photo-polymerisation process at layer thicknesses of 25, 50, and 100 μm. These taggants can be reliably detected using both optical methods and XCT. The combination of the physical and digital hashes generated in this study produces a robust “double-lock” system that demonstrates substantial spatial complexity and resistance to physical replication. Even assuming an adversary has full access to the verification data (XCT coordinates and colour codes) and identical raw materials, perfectly replicating the microscopic spatial arrangement of particles freely suspended in a liquid photopolymer during the layer-by-layer VPP curing process remains physically infeasible, thereby securing the system against physical spoofing.

XCT analysis revealed that the quantity of taggants within each sample is highly dependent on the chosen layer thickness, which concurrently influences their spatial distribution. The distribution of taggants at a low layer thickness (25 μm) was found to exhibit complete spatial randomness; however, it transitions to a non-random, clustered distribution at higher thicknesses, demonstrating reproducible micro-clustering in the 100 μm samples. This indicates that the implementation of these taggants must carefully account for the intended layer thickness, given its significant impact on both taggant volume and spatial dispersion.

Experimental evidence indicates that the embedded taggants do not affect the thermal properties of the resin components, primarily due to their low overall concentration. While the presence of taggants did induce a slight reduction in mechanical strength, this decrease was limited to less than 5% at both ambient and elevated temperatures (up to 220 °C). Furthermore, the concentration of embedded taggants directly correlates with the final mechanical properties, with lower concentrations resulting in minimal degradation of structural integrity.

By utilising cost-effective and commercially available taggants, this research provides a foundational proof of concept for future scalable systems of anti-counterfeiting measures within the manufacturing supply chain. The integration of these PUFs necessitated neither printer modifications nor specialised operational skills, presenting a highly accessible approach for manufacturers. While the taggants themselves demonstrated excellent resilience to environmental stressors, such as elevated temperatures, the current reliance on XCT equipment and specialised personnel to generate and decode the digital hash adds to operational costs and lead times. Consequently, future work should focus on mitigating this dependency by selectively embedding taggants near component surfaces to facilitate the use of portable, rapid-detection methodologies. While this study successfully establishes the foundational proof of concept for the “double-lock” PUF architecture using a single commercial platform and transparent resin to facilitate initial metrological benchmarking, scaling this technology will require rigorous future validation. To standardise calibration protocols and facilitate full-scale industrial deployment across diverse VPP ecosystems, subsequent research must encompass multi-machine reproducibility, the transition to fully opaque matrix materials, quantitative collision probability modelling, and empirical adversarial replication trials to confirm the system’s ultimate cryptographic maturity. Future investigations will also need to address cross-platform interoperability, validating the end-user verification process across micro-XCT equipment from varying manufacturers to ensure the authentication algorithm is robust against differing hardware calibrations and imaging artifacts.

## Data Availability

Data sets generated during the current study are available from the corresponding author on reasonable request.
